# Light-Activated Monomethyl Auristatin E Prodrug Nanoparticles for Combinational Photo-Chemotherapy of Pancreatic Cancer

**DOI:** 10.3390/molecules27082529

**Published:** 2022-04-14

**Authors:** In Kyung Cho, Man Kyu Shim, Wooram Um, Jong-Ho Kim, Kwangmeyung Kim

**Affiliations:** 1 Laboratory Animal Resource Center, Korea Research Institute of Bioscience and Biotechnology, Cheongju 28116, Korea; cik@kribb.re.kr; 2 Biomedical Research Institute, Korea Institute of Science and Technology, Seoul 02792, Korea; mks@kist.re.kr; 3 Department of Pharmaceutical Science, College of Pharmacy, Kyung Hee University, 26 Kyungheedae-ro, Dongdaemun-gu, Seoul 02447, Korea; jonghokim@khu.ac.kr; 4 School of Chemical Engineering, College of Engineering, Sungkyunkwan University, Suwon 16419, Korea; tings0609@nate.com; 5 KU-KIST Graduate School of Converging Science and Technology, Korea University, Seoul 02841, Korea

**Keywords:** pancreatic cancer, prodrug nanoparticles, combination treatment, synergistic effect

## Abstract

Pancreatic cancer is a highly fatal disease that is becoming an increasingly leading cause of cancer-related deaths. In clinic, the most effective approach to treat pancreatic cancers is the combination treatment of several chemotherapeutic drugs, including fluorouracil, leucovorin, irinotecan, and oxaliplatin (FOLFIRINOX), but this approach is not adequate to manage patients due to their severe toxic side effects. Herein, we proposed light-activated monomethyl auristatin E (MMAE) prodrug nanoparticles for combinational photo-chemotherapy and optimized its applications for pancreatic cancer treatment. The photosensitizer (Ce6) and chemotherapeutic drug (MMAE) were conjugated through caspase-3-specific cleavable peptide (KGDEVD). The resulting CDM efficiently promoted the reactive oxygen species (ROS) under visible light irradiation and thereby induced caspase-3 overexpression in pacreatic cancers, which subsequently released the MMAE from the system. Importantly, MMAE released from CDM further amplified the activation of CDM into MMAE by inducing extensive apoptotic cell death in tumor microenvironment for treatment of tumor cells in deep in the tumor tissues as far visible light cannot reach. In addition, CDM formed prodrug nanoparticles via intermolecular π-π stacking and hydrophobic interactions, allowing durable and reliable treatment by preventing fast leakage from the pancreatic cancers via the lymphatic vessels. The CDM directly (intratumoral) injected into pancreatic cancers in orthotopic models through an invasive approach significantly delayed the tumor progression by combinational photo-chemotherapy with less toxic side effects. This study offers a promising and alternative approach for safe and more effective pancreatic cancer treatment via prodrug nanoparticles that combine photodynamic therapy and chemotherapy.

## 1. Introduction

Pancreatic cancer, with a 5-year survival rate of less than 5%, is a highly fatal disease that is becoming an increasingly leading cause of cancer-related deaths [1]. Most patients are diagnosed with unresectable or metastatic disease; thus, their treatment options are limited [2,3]. In addition, even for the small subset of patients who have resectable pancreatic cancers, the prognosis remains very poor with only 20% survival rate of 5 years after surgery [4]. In the past decades, significant advances have been made in diagnostic methods, perioperative management, and local or systemic therapies for advanced malignancies, but there is only modest incremental progress in patient survival [5]. The most effective approach to treat advanced pancreatic cancers in patients is the combination treatment of several chemotherapeutic drugs, including fluorouracil, leucovorin, irinotecan, and oxaliplatin (FOLFIRINOX), which led to significantly prolonged survival of patients [6,7]. However, systemic administration of such drugs has risk of the severe toxic side effects due to their low cancer specificity; thus, there is still a desperate need for new treatment options that have a potent antitumor therapeutic potential with less side effects for safe and more effective pancreatic cancer treatment [8].

Photodynamic therapy (PDT), a mature localized phoththerapy with approval for clinical use in 1993, is an alternative treatment option for advanced cancers [9]. Under visible light irradiation, the photosensitizers absorb a photon of the light and released ions and energy is directly transferred to the molecular oxygen to produce a reactive oxygen species (ROS), such as singlet oxygen (^1^O_2_), superoxide (O_2_^−^), and peroxide anions (O_2_^2−^), which damage the cancer tissues [10]. However, limited penetration depth of visible light to biological tissues and oxygen depletion during treatment greatly reduce the antitumor therapeutic potential of PDT [11,12]. Therefore, it is hard to expect great outcomes of patients for complete regression of pancreatic cancer by monotherapy of PDT. To overcome these obstacles, PDT can be combined with chemotherapeutic drugs to expect synergistic effect for improved therapeutic efficacy in pancreatic cancer; thus, developing a promising drug to combine PDT with a chemotherapeutic drug for combinational photo-chemotherapy and optimizing its treatment methods for pancreatic cancer are formidable challenges [13,14].

**Figure 1:**
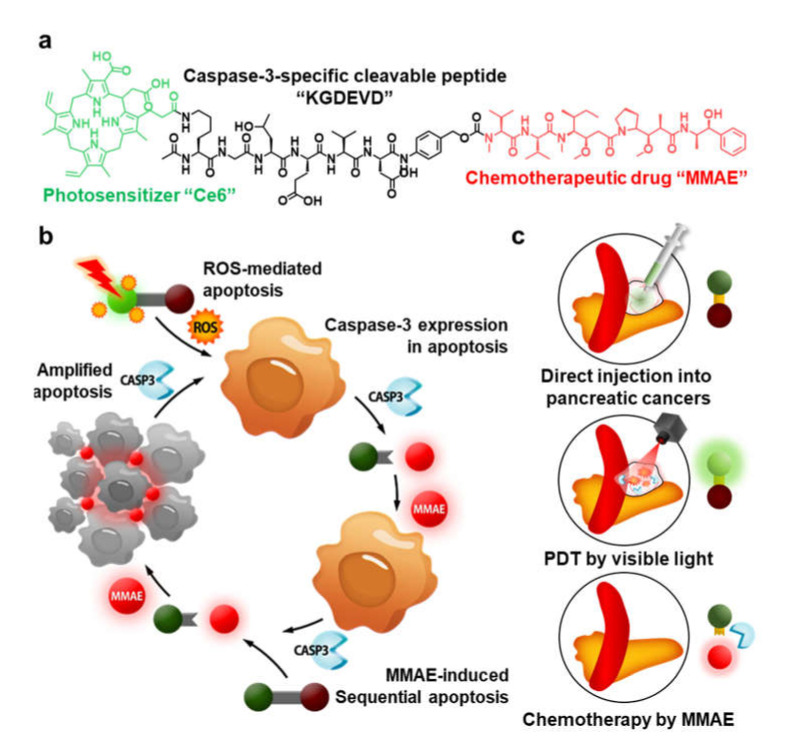
**Light-activated monomethyl auristatin E (MMAE) prodrug nanoparticles and its application for pancreatic cancer treatment.** (**a**) Photosensitizer (Ce6) and chemotherapeutic drug (MMAE) are chemically conjugated through the caspase-3-specific cleavable KGDEVD peptide, resulting in CDM. (**b**) The CDM promotes a significant reactive oxygen species (ROS) in cancer cells and thereby induces caspase-3 overexpression by apoptosis, which subsequently triggers release of MMAE. (**c**) Directly injected CDM in pancreatic cancers causes a potent antitumor therapeutic potential by combinational photo-chemotherapy under visible light irradiation, with less toxic side effects.

Herein, we proposed light-activated monomethyl auristatin E (MMAE) prodrug nanoparticles, constructed with photosensitizer chorin e6 (Ce6), caspase-3-specific cleavable KGDEVD peptide, and a chemotherapeutic drug (MMAE) for combinational photo-chemotherapy of pancreatic cancer. The MMAE was chemically conjugated to C-terminus of KGDEVD peptide via the self-immolative linker (PABC; *p*-aminobenzylcarbamate), and Ce6 was further introduced to N-terminus of peptide (Figure 1a). The resulting Ce6-KGDEVD-PABC-MMAE prodrug (CDM) promotes a significant ROS by visible light-induced PDT, and overexpressed caspase-3 in tumor tissues by apoptosis, subsequently trigger release of MMAE by enzymatic cleavage of KGDEVD peptide (Figure 1b). Importantly, MMAE released from CDM further amplified the activation of CDM into MMAE by inducing extensive apoptotic cell death in the tumor microenvironment for treatment of tumor cells deep in the tissues as far visible light cannot reach. Compared with the efficacy of monotherapy of PDT that is restricted by depletion of oxygen during treatment and depth limitation of light, CDM induced considerable cytotoxic effects by releasing additionally MMAE that has 100–1000 times higher potency than doxorubicin [8]. In contrast, the release of MMAE from CDM is greatly minimized in the absence of visible light due to lack of caspase-3 expression, thereby reducing the risk of the severe toxic side effects during treatment. Interestingly, CDM form stable nanoparticles via the intermolecular π-π stacking and hydrophobic interactions, which prevent fast leakage from the tumors via the lymphatic vessels to allow durable and reliable treatment [15,16,17,18]. In this study, we established the optimal treatment methods for pancreatic cancers using the CDM-mediated photo-chemotherapy. The CDM is directly (intratumoral) injected into pancreatic cancers in orthotopic models through invasive approach, and cancer tissue is locally irradiated by visible light to promote the caspase-3 overexpression by inducing apoptosis, resulting in subsequent release of MMAE (Figure 1c). This study demonstrated a promising therapeutic potential of combinational photo-chemotherapy by CDM in orthotopic pancreatic cancer models compared with monotherapy of photodynamic therapy or chemotherapy.

## 2. Results and Discussion

### 2.1. Preparation and Characterization of CDM

**Figure 2:**
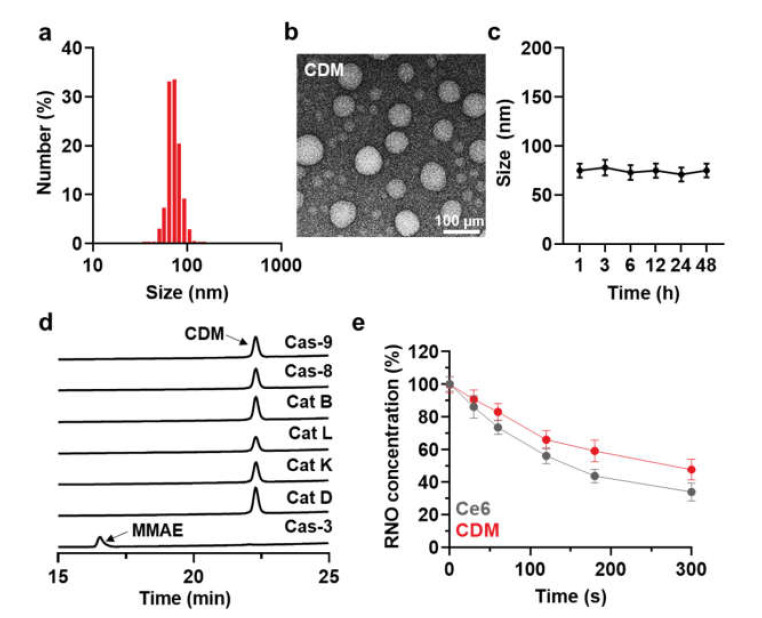
**Physicochemical characterization of CDM.** (**a**) Size distribution of CDM in aqueous condition (saline; 1 mg/kg). (**b**) TEM image showing spherical morphology of CDM. (**c**) Stability of CDM nanoparticles in saline. (**d**) Target enzyme-specific cleavage of CDM was assessed after incubation with caspase-3 and various other enzymes. (**e**) ROS generation of CDM or Ce6 in the presence of visible light irradiation.

As an alternative therapeutic approach to combine PDT and chemotherapy for pancreatic cancer treatment, light-activated monomethyl auristatin E (MMAE) prodrug was prepared by conjugating photosensitizer (Ce6), caspase-3-specific cleavable peptide (KGDEVD), and a chemotherapeutic drug (MMAE). The MMAE was conjugated to the C-terminus of KGDEVD peptide through a self-immolative linker (PABC), and Ce6 was further introduced to the N-terminus of peptide via EDC/NHS reaction, resulting in CDM (Figure S1). The target enzyme of caspase-3 is the most widely studied as a biomarker for cancer-specific diagnosis and therapy, as it plays a key role in both the intrinsic and extrinsic death receptor pathways [19]. The KGDEVD peptide was used as an apoptosis-specific cleavable linker because it has great responsiveness to active caspase-3 at an early stage of apoptosis [20]. In addition, the self-immolative linker, PABC, was selected to design the prodrug owing to its favorable electronic and steric characteristics for enzymatic activation [21]. Finally, the MMAE that has 100–1000 times higher potency than doxorubicin was conjugated to amplify the antitumor therapeutic potential by subsequent release after visible light irradiation for PDT [8]. After the reaction, 99% of CDM was purified with high-performance liquid chromatography (HPLC; Figure S2). The molecular weight of CDM was also measured using a MALDI-TOF mass spectrometer, wherein the exact molecular weight was calculated to be 2131.1 Da and then confirmed to be 2131.1 *m*/*z* (Figure S3). Interestingly, CDM efficiently self-assembled into prodrug nanoparticles via intermolecular π-π stacking and hydrophobic interactions, showing a size distribution of 50–110 nm with an average size of 75.1 ± 4.4 nm in aqueous condition (Figure 2a) [15,16,17]. A TEM image further showed the spherical structure of CDM nanoparticles in saline (Figure 2b). In addition, CDM nanoparticles showed great stability in saline; significant changes of particle size were not observed for 48 h of incubation (Figure 2c). These nano-sized particles of CDM can prevent the rapid clearance through lymphatic vessels from the pancreatic cancers, which allow more durable and reliable treatment compared with small molecular drugs [18]. Next, we assessed the target enzyme-specific cleavage of CDM nanoparticles. When the CDM nanoparticles were incubated with caspase-3 for 24 h, approximately 99% of CDM was cleaved, resulting in release of MMAE (Figure 2d). In contrast, CDM nanoparticles were not cleaved after incubation with caspase-9, caspase-8, cathepsin B, cathepsin L, cathepsin K, and cathepsin D for 24 h, indicating high target enzyme-specificity. Upon visible light irradiation, the ROS production from CDM was nearly similar with Ce6 in same experimental conditions (Figure 2e). The photophysical property of CDM was further confirmed by the Singlet Oxygen Sensor Green (SOSG) assays, wherein efficiency for singlet oxygen generation of CDM was similar with Ce6 (Figure S4). This result demonstrated that chemical modification with KGDEVD-MMAE did not affect the ability to promote ROS under visible light irradiation of photosensitizer, Ce6. Taken together, CDM efficiently formed prodrug nanoparticles with photosensitizer and chemotherapeutic drug, and it was expected that they promote a significant ROS in pancreatic cancers under visible light irradiation to induce caspase-3 overexpression, which subsequently release the MMAE.

### 2.2. In Vitro Cytotoxicity and Caspase-3 Overexpression by CDM

**Figure 3:**
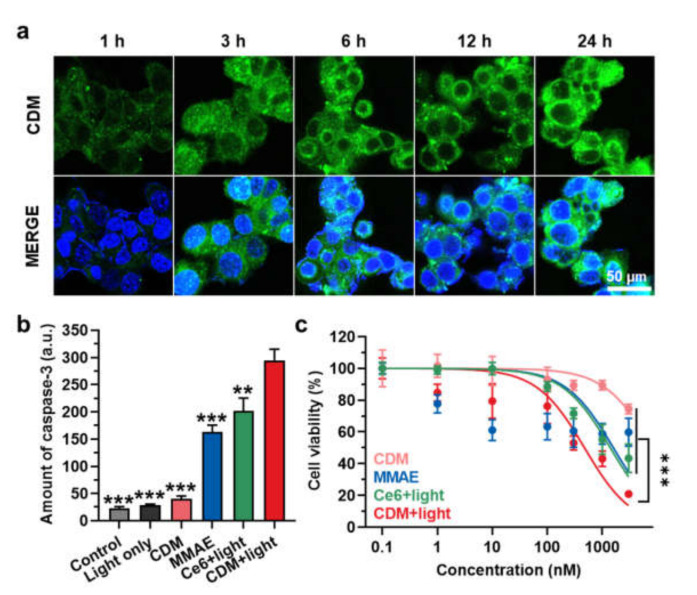
**In vitro cytotoxicity and caspase-3 overexpression by CDM.** (**a**) Time-dependent cellular uptake of CDM in human pancreatic cancer cell, KPC960. (**b**) The caspase-3 expression levels in KPC960 cells after treatment of CDM, MMAE, or Ce6. CDM- or Ce6-treated KPC960 cells were exposed to visible light with power of 40 mW for 5 min. The asterisks in Figure indicate the comparison to the CDM+light group. (**c**) The viability of KPC960 cells after treatment of CDM, MMAE, or Ce6. CDM- or Ce6-treated KPC960 cells were exposed to visible light with power of 40 mW for 5 min.

We assessed in vitro cytotoxicity and caspase-3 overexpression by CDM under visible light irradiation. First, we found the optimal irradiation timing by evaluating cellular uptake of CDM over time after incubation in human pancreatic cancer cell line KPC960. When the CDM (500 nM) was incubated with KPC960, the fluorescence (Ex/Em:633/660) of CDM was gradually increased in an incubation time-dependent manner, and that was saturated after 12 h of incubation (Figure 3a). Then, the caspase-3 expression levels in the KPC960 cells were assessed after treatment of MMAE, Ce6 with visible light, or CDM with visible light (Figure 3b). The KPC960 cells were incubated with an equivalent dose (500 nM) of MMAE, Ce6, or CDM for 12 h, and Ce6- or CDM-treated KPC960 cells were exposed to visible light with power of 40 mW for 5 min. As expected, MMAE- or Ce6 (with light)-treated KPC960 cells showed significantly elevated amounts of caspase-3 due to apoptosis induction of cells compared with the control group. Notably, CDM with light-treated KPC960 expressed the highest amount of caspase-3, which was significantly upregulated compared with a single treatment of Ce6 (with light) and MMAE. These results suggested that caspase-3 overexpression during PDT by CDM subsequently promotes the release of MMAE, thus amplifying apoptosis of pancreatic cancer cells. As a control, caspase-3 expression in KPC960 was not upregulated after treatment with CDM in the absence of visible light irradiation; thus, we expected that CDM can reduce toxic side effects during treatment by restricting the drug activation in off-target sites. Finally, combinational photo-chemotherapy of CDM resulted in a potent cytotoxicity in KPC960 cells. The viability of KPC960 cells were assessed after treatment with MMAE, Ce6 with light, or CDM with or without light for 12 h (Figure 3c). As expected, CDM in the presence of light showed significantly higher cytotoxicity in KPC960 cells compared with MMAE and Ce6 with light, which further confirmed the enhanced antitumor therapeutic potential of combinational photo-chemotherapy.

As a control, the cytotoxicity of CDM in cardiomyocytes (H9C2) and human dermal fibroblasts (HDF) was also assessed in absence of visible light to confirm whether CDM can prevent non-specific cleavage in the off-target tissues for minimizing severe toxic side effects. As expected, significant cytotoxicity was not observed in both cell lines (Figure S5). These in vitro results clearly demonstrated the mode of action (MOA) of CDM that promotes caspase-3 overexpression in the pancreatic cancer cells by Ce6-mediated PDT and the subsequently release of the MMAE for synergistic effects.

### 2.3. In Vivo Therapeutic Efficacy of CDM in Orthotopic Pancreatic Cancer Models

**Figure 4:**
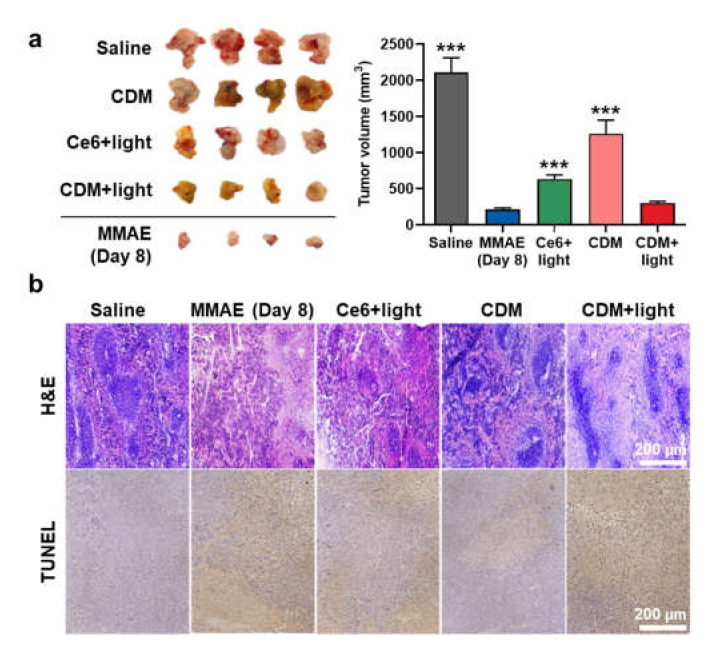
**In vivo therapeutic efficacy of CDM in orthotopic pancreatic cancer models.** (**a**) Optical images and volume of cancer tissues from orthotopic pancreatic cancer models after treatment with saline, MMAE, Ce6, or CDM. The tumors of Ce6- or CDM-treated mice were locally irradiated with visible light with power of 160 mW for 10 min. The asterisks in Figure indicate the comparison to the CDM+light group. (**b**) Pancreatic cancer tissues stained with H&E or TUNEL.

We next evaluated a therapeutic efficacy of combinational photo-chemotherapy by CDM in orthotopic pancreatic cancer models. The mice models were prepared by direct inoculation of KPC960 cells (1 × 10^5^) into the pancreas tissue after incision at the left abdominal side. After 14 days of inoculation, the mice were randomly divided into five groups of saline, MMAE, Ce6 with light, or CDM with or without light, and each drug (0.1 mg/kg of Ce6 and MMAE, 0.3 mg/kg of CDM as equivalent dose of 0.1 mg/kg of MMAE) was directly injected into pancreatic cancers via the invasive approach. In case of Ce6 or CDM groups, pancreatic cancer tissues were locally irradiated with power of 160 mW for 10 min. The visible light irradiation was performed after minimum incision, as shown in Figure S6. Importantly, CDM with light (299.31 ± 22.1 mm^3^) significantly delayed the growth of pancreatic cancer compared with saline (2105.11 ± 205.1 mm^3^), Ce6 with light (631.51 ± 60.22 mm^3^), and CDM without light (1261.21 ± 188.31 mm^3^) groups on day 15 after treatments (Figure 4a). The mice treated with MMAE showed delayed progression of pancreatic cancer (211.21 ± 20.51 mm^3^ on day 8), but they were all dead within 8 days of treatment due to severe toxic side effects of MMAE. The pancreatic cancer tissues stained with H&E or TUNEL exhibited the elevated damaged areas in CDM with the light group compared with other treatments, which further confirmed the enhanced therapeutic efficacy of CDM-mediated combinational photo-chemotherapy (Figure 4b). Taken together, these in vivo results demonstrated that the combination of Ce6-mediated PDT and MMAE-mediated chemotherapy by CDM induces a potent therapeutic efficacy in the pancreatic cancers.

### 2.4. The Safety of CDM Treatment in Orthotopic Pancreatic Cancer Models

**Figure 5:**
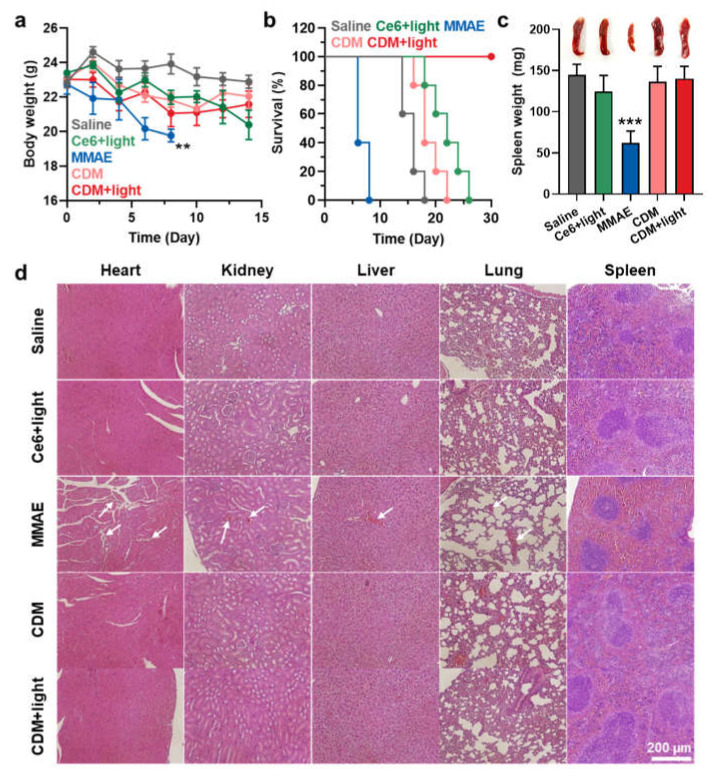
**The safety of CDM treatment in orthotopic pancreatic cancer models.** (**a**) The body weight change during treatment with saline, MMAE, Ce6, or CDM. (**b**) Mice survival during treatment with saline, MMAE, Ce6, or CDM. (**c**) The spleen weights of orthotopic pancreatic cancer models after 8 days of each treatment. (**d**) The organ tissues stained with H&E after 8 days of each treatment. The asterisks in Figure indicate the comparison to the CDM+light group.

The safety of CDM treatment was assessed in orthotopic pancreatic cancer models, which were treated as same protocol in Figure 4. First, mice treated with saline, Ce6 with light, or CDM with or without light did not show significant body weight changes during treatments (Figure 5a). In contrast, MMAE treatment resulted in significant body weight loss of mice due to their severe toxicity, and mice were eventually dead within 8 days after treatment (Figure 5b). The median survival of the mice treated with saline, Ce6 with light, and CDM without light was determined to be 15 days, 24 days, and 18 days, wherein the mice were dead owing to the tumor progression. On the contrary, mice in the CDM with light group survived over 30 days by delaying tumor growth and reducing toxic side effects. The safety of CDM treatment was further evaluated by analyzing normal organ tissues on day 8 after treatment. As expected, since the spleen is located nearby pancreatic cancer, MMAE-treated mice revealed a significant reduction in spleen weight (Figure 5c). However, CDM greatly reduced the MMAE-mediated toxicity during treatment by releasing MMAE specifically under visible light irradiation that promotes caspase-3 expression and minimizing the drug leakage from the pancreatic cancers via the nano-sized particle.

As a result, the spleen weight of mice treated with CDM was nearly similar to that of saline-treated mice. In addition to spleen tissue, other tissues were also stained with H&E to observe damaged areas on day 8 after treatments (Figure 5d). Importantly, MMAE treatment resulted in extensive toxicity in every organ (white arrows), but CDM-treated mice showed only negligible structural abnormalities in the organ tissues. These results showed that even with the 100–1000 times more potent efficacy than doxorubicin, the clinical use of MMAE is strictly hindered owing to the severe toxicity, but CDM efficiently mitigated MMAE-related side effects based on prodrug nanoparticle formulation that can be specifically activated by overexpressed caspase-3 at defined tumor tissues under visible light irradiation.

## 3. Materials and Methods

### 3.1. Materials

Ac-K(Alloc)GD(All)E(All)VD(All)-OH (alloc-KGDEVD) was purchased from Peptron (Daejeon, Korea). Chlorin e6 (Ce6) was purchased from Frontier Scientific Inc. (Logan, UT, USA). 2-ethoxy-1-ethoxycarbonyl-1,2-dihydroquinoline (EEDQ), Bis(p-nitrophenyl) carbonate, 1-ethyl-3(3-dimethylaminopropyl) carbodiimide (EDC), Dimethyl sulfoxide (DMSO), *N*,*N*-diisopropylethylamine (DIPEA), hydroxybenzotriazole (HOBt), p-aminobenzyl alcohol, tetrakis(triphenylphosphine)palladium, and N-hydroxysuccinimide (NHS) were purchased from Sigma Chemical Co. (St. Louis, MO, USA). Anhydrous dimethylformamide (DMF) and dimethylsulfoxide (DMSO) were purchased from Merck (Darmstadt, Germany). Tributyltin hydride (Bu3SnH) and glacial acetic acid were purchased from Acros (St. Louis, MO, USA). DMEM medium, penicillin–streptomycin, and fetal bovine serum (FBS) were purchased from WelGENE Inc. (Daegu, Korea). Cathepsin B, D, K, L, caspase-9, caspase-3, and Caspase-3 ELISA Kit (cat# SMIF00) were purchased from R&D system (Minneapolis, MN, USA). KPC960 (Human pancreatic cancer) cell lines were purchased from American Type Culture Collection (ATCC; Manassas, VA, USA). Tem grid (Carbon Film 200 Mesh copper) was purchased from Electron Microscopy Sciences (Hatfield, PA, USA). Cell counting kit-8 (CCK-8) was purchased from Vitascientific (Beltsville, MD, USA).

### 3.2. Preparation of CDM Nanoparticles

Briefly, alloc-KGDEVD (0.5 g, 0.55 mmol), 4-aminobenzyl alcohol (0.34 g, 1.1 mmol, 1 eq.) and 2-Ethoxy-1-ethoxycarbonyl-1,2-digydroquinoline (0.135 g, 1.1 mmol, 1 eq.) were reacted in anhydrous *N*,*N*-Dimethylformamide (15 mL) overnight in room temperature. The solution was added into diethyl ether to from dried powder. Then, the obtained chemicals were further incubated in *N*,*N*-Dimethylformamide (25 mL) with bis(p-nitrophenyl)carbonate (5 eq.) and *N*,*N*-Diisopropylethylamine (3 eq.) at room temperature for 1 h. The mixed solution was precipitated in diethyl ether. The dried sediment (300 mg), MMAE (250 mg, 0.6 eq.), and 1-Hydroxybenzotriazole hydrate (30 mg, 0.75 eq.) were mixed in anhydrous DMF (20 mL). After 1 h of reaction, azabenzene (5 mL) and *N*,*N*-Diisopropylethylamine (100 μL, 1 eq.) were further added and mixed for 72 h at room temperature. For the removal of protection groups, Alloc-KGDEVD-PABC-MMAE was reacted with tributyltin hydride (10 eq.), tetrakis(triphenylphosphine)palladium (1 eq.), and acetic acid (20 eq.) under nitrogen gas atmosphere for 2 h. Then, the KGDEVD-PABC-MMAE (1 eq.) was reacted with Ce6 (1 eq.) in the presence of EDC (3 eq.) and NHS (3 eq.). After 24 h of reaction, the resulting Ce6-(Ac)KGDEVD-PABC-MMAE was purified using the C18 reverse analytical column. After preparation, the purity was analyzed by the reverse-phase HPLC system.

### 3.3. Characterization of CDM Nanoparticles

The size distribution of CDM in saline (1 mg/mL) was characterized by using dynamic light scattering (DLS; SZ-100, Horiba. Ltd., Kyoto, Japan, 532 nm, 10 mW). The morphology of CDM nanoparticles in saline (1 mg/mL) was observed using the transmission electron microscope (TEM; Talos F200X; FEI Compant, Hillsboro, OR, USA) after negative staining with 1% uranyl acetate. The ROS generation was assessed by bleaching test using p-nitroso-N,N’dimethylaniline (RNO). The Ce6 and Ce6-DEVD-MMAE (1 μM) was dissolved in saline containing 1.2 mM of L-histidine and 1% DMSO with 10 μM of RNO. Then, the samples were irradiated with visible light (Shanghai Dream Laser Technology Co., Ltd., Shanghai, China) with power of 40 mW for 5 min. Finally, the RNO absorbance was measured via UV-vis spectrometer (Agilent Spectroscopy System, Agilent Technology, Santa Clara, CA, USA) at 405 nm of each sample (n = 5). In the same conditions, the photophysical property of CDM was further confirmed by the Singlet Oxygen Sensor Green (SOSG) assays. The Ce6 and Ce6-DEVD-MMAE (1 μM) were mixed with SOSG (0.25 μM). Then, solutions were irradiated with visible light with power of 40 mW for different time periods, and the fluorescence intensity was measured using a fluorescence spectrometer (Hitachi, Tokyo, Japan). The caspase-3-specific cleavage of CDM nanoparticles was analyzed by reverse-phase HPLC system (Agilent Technologies 1200 series, Agilent Technologies, USA). The CDM nanoparticles (10 μM) were incubated with caspase-3, caspase-9, caspase-8, cathepsin B, cathepsin L, cathepsin K, or cathepsin D (10 μg) in buffer (0.1% CHAPS, 0.9% NaCL, 50 mM HEPES, 1 mM EDTA, 10 mM DTT, 10% glycerol, pH 7.6) for 24 h at 37 °C.

### 3.4. In Vitro Cellular Uptake of CDM Nanoparticles

To observe the cellular uptake of CDM nanoparticles, 3 × 10^5^ KPC960 cells were seeded into 35-mm confocal dishes with DMEM media. Then, the cells were treated with 10 μM of CDM nanoparticles for 1, 3, 6, 12, and 24 h, respectively. After treatment, all cells were fixed with 2% paraformaldehyde solution for 10 min and stained with 4,6-diamidino-2-phenylindole (DAPI) for 15 min. The fluorescence imaging was performed using a confocal laser microscope (Leica TCS SP8, Leica Microsystems GmbH, Wetzlar, Germany).

### 3.5. Quantitative Assay of Caspase-3 Expression

To assess the caspase-3 expression after each treatment, the KPC960 cells were incubated with an equivalent amount (500 nM) of MMAE, Ce6 or CDM. In case of Ce6 or CDM groups, the cells were exposed to visible light with power of 40 mW for 5 min. After treatment, amount of caspase-3 in the cell lysates were measured using the caspase-3 assay kit.

### 3.6. In Vitro Cytotoxicity of CDM Nanoparticles

The cytotoxicity of CDM nanoparticles was evaluated via the cell counting kit-8. The KPC960 cells (5 × 10^4^) were seeded in a 96-well cell culture plates. After 24 h of stabilization, the cells were treated with various concentrations of Ce6, MMAE, or CDM nanoparticles for 24 h. The cells treated with Ce6 or CDM were irradiated with visible light (40 mW for 5 min) after 6 h of drug treatment. Then, 200 μL of medium containing 10% CCK-8 solution was added in each well, and cell viability was measured by a microplate reader (VERSAmac^TM^; Molecular Devices Corp., San Jose, CA, USA) (n = 5). As a control, cell viability of H9C2 and HDF after treatment with CDM nanoparticles for 24 h in absence of visible light irradiation was assessed.

### 3.7. Preparation of Orthotopic Pancreatic Cancer Models and Treatment Protocol

Mice were bred and maintained under specific pathogen-free conditions at the Korea Institute of Science and Technology (KIST). All experiments were conducted using protocols approved by the Association for Assessment and Accreditation of Laboratory Animal Care at the KIST. The BALB/C nude mice were anesthetized with Zoletil (8%), Ketamine (2%), and the left abdominal side was incised. Then, KPC960 cells (1 × 10^5^) suspended in 10 μL saline were directly injected into the pancreas. The ventral wound was sutured in one layer with 6-0 non-absorbable sutures (Ailee Co., Busan, Korea). After 14 days of inoculation, mice were randomly divided into five groups (n = 4); (i) Saline (10 μL); (ii) Ce6 (0.1 mg/kg in 10 μL of saline) with visible light; (iii) MMAE (0.1 mg/kg in 10 μL of saline); (iv) CDM (0.3 mg/kg in 10 μL of saline); or (v) CDM (0.3 mg/kg in 10 μL of saline) with visible light. Each drug was directly injected into pancreatic cancers after incision at the left abdominal side, and tumors of Ce6 and CDM nanoparticle groups were irradiated with visible light with power of 160 mW for 10 min. The weight was measured every 2 days until 2 weeks after post-injection and the animals ware sacrificed. The tumor was fixed with 4% paraformaldehyde solution for paraffin embedding.

### 3.8. Statistics

The statistical significance between two groups was analyzed using Student’s t-test. One-way analysis of variance (ANOVA) was performed for comparisons of more than two groups, and multiple comparisons were analyzed using Tukey–Kramer post-hoc test. Statistical significance is indicated with an asterisk (* *p* < 0.05, ** *p* < 0.01, and *** *p* < 0.001) in the figures.

## 4. Conclusions

In this study, we proposed light-activated monomethyl auristatin E (MMAE) prodrug nanoparticles and optimized their application for pancreatic cancer treatment. The prodrug nanoparticles (CDM) were prepared by self-assembly of photosensitizer (Ce6), caspase-3-specific cleavable peptide (KGDEVD), and MMAE conjugates. The CDM could promote ROS under visible light irradiation and thereby induce caspase-3 overexpression in cancer cells, which subsequently triggered the release of MMAE. When the CDM was directly injected into pancreatic cancers of orthotopic models with visible light irradiation, the progression of cancers was significantly delayed by a potent therapeutic efficacy of combinational Ce6-mediated PDT and MMAE-mediated chemotherapy, compared with a single treatment of PDT or MMAE. In particular, CDM also greatly reduced the MMAE-related severe toxic side effects by restricting the drug activation in off-target tissues. Overall, these results suggested that combinational photo-chemotherapy by light-activated MMAE prodrug nanoparticles provides a promising and alternative therapeutic approach for pancreatic cancer.

## Data Availability

Not applicable.

## Supplementary Materials

The following supporting information can be downloaded at: https://www.mdpi.com/article/10.3390/molecules27082529/s1, Figure S1: Synthetic route to prepare light-activated MMAE prodrugs (CDM). Figure S2: The purity of CDM after preparation, confirmed via the HPLC. Figure S3: The exact molecular weight of CDM was calculated to be 2131.1 Da and measured to be 2131.1 *m*/*z* [M], which was confirmed via the MALDI-TOF mass spectrometer. Figure S4: Singlet oxygen generation from Ce6 and CDM under visible light irradiation, as con-firmed via the Singlet Oxygen Sensor Green (SOSG) assays. Figure S5: The viability of H9C2 and HDF cells after treatment of CDM in absence of visible light. Figure S6: The photos to show the procedure of visible light irradiation for in vivo experiments.

## Sample Availability

Samples of the compounds CDM are available from the authors.

## References

[1] Hidalgo M. (2010). Pancreatic Cancer. N. Engl. J. Med..

[2] Kamisawa T., Wood L.D., Itoi T., Takaori K. (2016). Pancreatic cancer. Lancet.

[3] Liu L., Kshirsagar P.G., Gautam S.K., Gulati M., Wafa E.I., Christiansen J.C., White B.M., Mallapragada S.K., Wannemuehler M.J., Kumar S. (2022). Nanocarriers for pancreatic cancer imaging, treatments, and immunotherapies. Theranostics.

[4] Bardeesy N., DePinho R.A. (2002). Pancreatic cancer biology and genetics. Nat. Rev. Cancer.

[5] Zhang L., Sanagapalli S., Stoita A. (2018). Challenges in diagnosis of pancreatic cancer. World J. Gastroenterol..

[6] Conroy T., Desseigne F., Ychou M., Bouché O., Guimbaud R., Bécouarn Y., Adenis A., Raoul J.-L., Gourgou-Bourgade S., de la Fouchardière C. (2011). FOLFIRINOX versus Gemcitabine for Metastatic Pancreatic Cancer. N. Engl. J. Med..

[7] Janssen Q.P., van Dam J.L., Doppenberg D., Prakash L.R., van Eijck C.H.J., Jarnagin W.R., O’Reilly E.M., Paniccia A., Besselink M.G., Katz M.H.G. (2022). FOLFIRINOX as Initial Treatment for Localized Pancreatic Adenocarcinoma: A Retrospective Analysis by the Trans-Atlantic Pancreatic Surgery Consortium. JNCI J. Natl. Cancer Inst..

[8] Kyu Shim M., Yang S., Sun I.-C., Kim K. (2022). Tumor-activated carrier-free prodrug nanoparticles for targeted cancer Immunotherapy: Preclinical evidence for safe and effective drug delivery. Adv. Drug Deliv. Rev..

[9] Yang S., Sun I.-C., Hwang H.S., Shim M.K., Yoon H.Y., Kim K. (2021). Rediscovery of nanoparticle-based therapeutics: Boosting immunogenic cell death for potential application in cancer immunotherapy. J. Mater. Chem. B.

[10] Dolmans D.E.J.G.J., Fukumura D., Jain R.K. (2003). Photodynamic therapy for cancer. Nat. Rev. Cancer.

[11] Bansal A., Yang F., Xi T., Zhang Y., Ho John S. (2018). In vivo wireless photonic photodynamic therapy. Proc. Natl. Acad. Sci. USA.

[12] Lee D., Kwon S., Jang S.-y., Park E., Lee Y., Koo H. (2022). Overcoming the obstacles of current photodynamic therapy in tumors using nanoparticles. Bioact. Mater..

[13] Um W., Park J., Ko H., Lim S., Yoon H.Y., Shim M.K., Lee S., Ko Y.J., Kim M.J., Park J.H. (2019). Visible light-induced apoptosis activatable nanoparticles of photosensitizer-DEVD-anticancer drug conjugate for targeted cancer therapy. Biomaterials.

[14] Choi J., Shim M.K., Yang S., Hwang H.S., Cho H., Kim J., Yun W.S., Moon Y., Kim J., Yoon H.Y. (2021). Visible-Light-Triggered Prodrug Nanoparticles Combine Chemotherapy and Photodynamic Therapy to Potentiate Checkpoint Blockade Cancer Immunotherapy. ACS Nano.

[15] Shim M.K., Park J., Yoon H.Y., Lee S., Um W., Kim J.-H., Kang S.-W., Seo J.-W., Hyun S.-W., Park J.H. (2019). Carrier-free nanoparticles of cathepsin B-cleavable peptide-conjugated doxorubicin prodrug for cancer targeting therapy. J. Control Release.

[16] Shim M.K., Moon Y., Yang S., Kim J., Cho H., Lim S., Yoon H.Y., Seong J.-K., Kim K. (2020). Cancer-specific drug-drug nanoparticles of pro-apoptotic and cathepsin B-cleavable peptide-conjugated doxorubicin for drug-resistant cancer therapy. Biomaterials.

[17] Moon Y., Shim M.K., Choi J., Yang S., Kim J., Yun W.S., Cho H., Park J.Y., Kim Y., Seong J.-K. (2022). Anti-PD-L1 peptide-conjugated prodrug nanoparticles for targeted cancer immunotherapy combining PD-L1 blockade with immunogenic cell death. Theranostics.

[18] Lim S., Park J., Shim M.K., Um W., Yoon H.Y., Ryu J.H., Lim D.-K., Kim K. (2019). Recent advances and challenges of repurposing nanoparticle-based drug delivery systems to enhance cancer immunotherapy. Theranostics.

[19] Shim M.K., Yoon H.Y., Lee S., Jo M.K., Park J., Kim J.-H., Jeong S.Y., Kwon I.C., Kim K. (2017). Caspase-3/-7-Specific Metabolic Precursor for Bioorthogonal Tracking of Tumor Apoptosis. Sci. Rep..

[20] Cho Y.S., Kim G.C., Lee H.M., Kim B., Kim H.R., Chung S.W., Chang H.W., Ko Y.G., Lee Y.S., Kim S.W. (2022). Albumin metabolism targeted peptide-drug conjugate strategy for targeting pan-KRAS mutant cancer. J. Control Release.

[21] Shim M.K., Yoon H.Y., Ryu J.H., Koo H., Lee S., Park J.H., Kim J.-H., Lee S., Pomper M.G., Kwon I.C. (2016). Cathepsin B-Specific Metabolic Precursor for In Vivo Tumor-Specific Fluorescence Imaging. Angew. Chem. Int. Ed..

