# Supplementary material for: Light-Activated Monomethyl Auristatin E Prodrug Nanoparticles for Combinational Photo-Chemotherapy of Pancreatic Cancer
Source: Molecules. 2022 Apr 14;27(8):2529. doi: 10.3390/molecules27082529 (PMC9032578; doi:10.3390/molecules27082529)
Supplement: Supplementary file 1 [file molecules-27-02529-s001.zip › molecules-1669277-supplementary.pdf]

Supplementary Information for

Light-Activated Monomethyl Auristatin E Prodrug Nanoparticles for Combinational Photo-Chemotherapy of Pancreatic Cancer

In Kyung Cho[1,2,3], Man Kyu Shim[2], Wooram Um[4], Jong-Ho Kim[3] and Kwangmeyung Kim[2,5,*]

[1]Laboratory Animal Resource Center, Korea Research Institute of Bioscience and Biotechnology, Cheongju 28116, Korea; cik@kribb.re.kr

[2]Biomedical Research Institute, Korea Institute of Science and Technology, Seoul 02792, Korea; mks@kist.re.kr

[3]Department of Pharmaceutical Science, College of Pharmacy, Kyung Hee University, 26 Kyungheedae-ro, Dongdaemun-gu, Seoul 02447, Korea; jonghokim@khu.ac.kr

[4]School of Chemical Engineering, College of Engineering, Sungkyunkwan University, Suwon 16419, Korea; tings0609@nate.com

[5]KU-KIST Graduate School of Converging Science and Technology, Korea University, Seoul 02841, Korea

[#]These authors contributed equally to this work.

*Correspondence and requests for materials should be addressed to **K. Kim** (E-mail: kim@kist.re.kr; address: Biomedical Research Institute, Korea Institute of Science and Technology (KIST), Seoul, 02792, Republic of Korea).

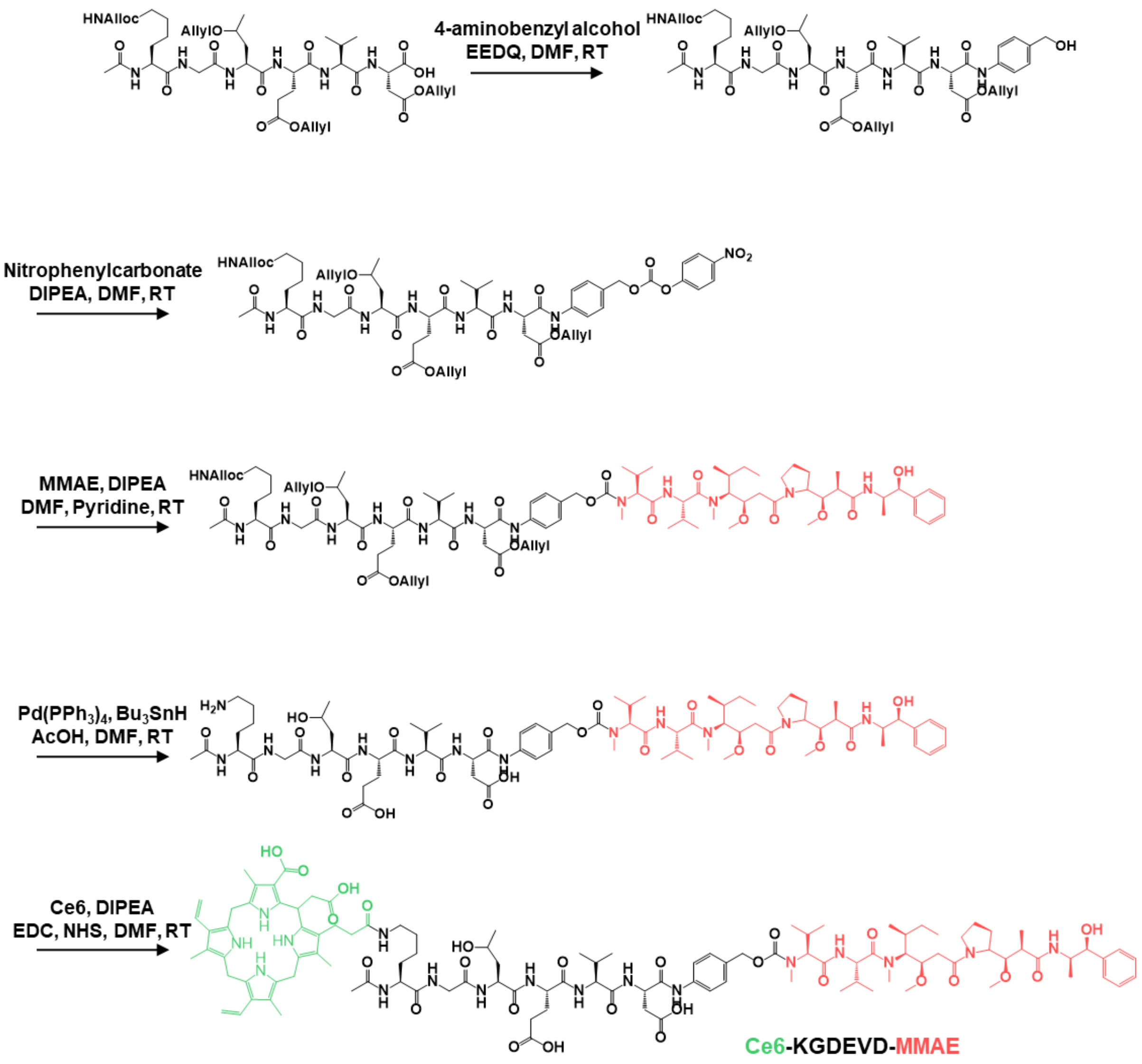

Figure S1. Synthetic route to prepare light-activated MMAE prodrugs (CDM).

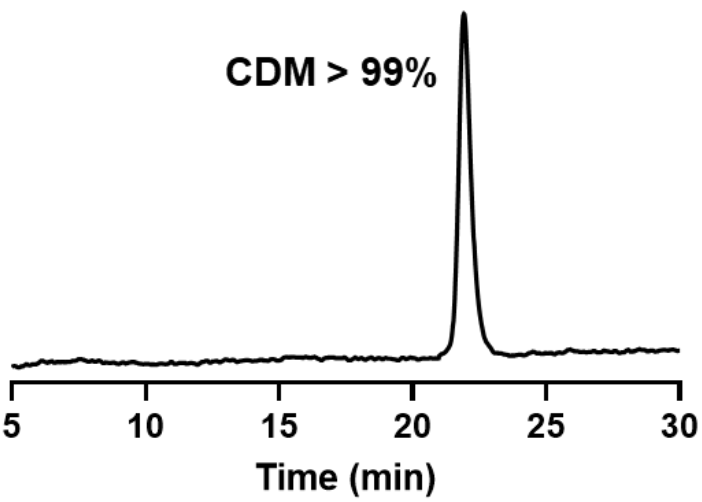

Figure S2. The purity of CDM after preparation, confirmed *via* the HPLC.

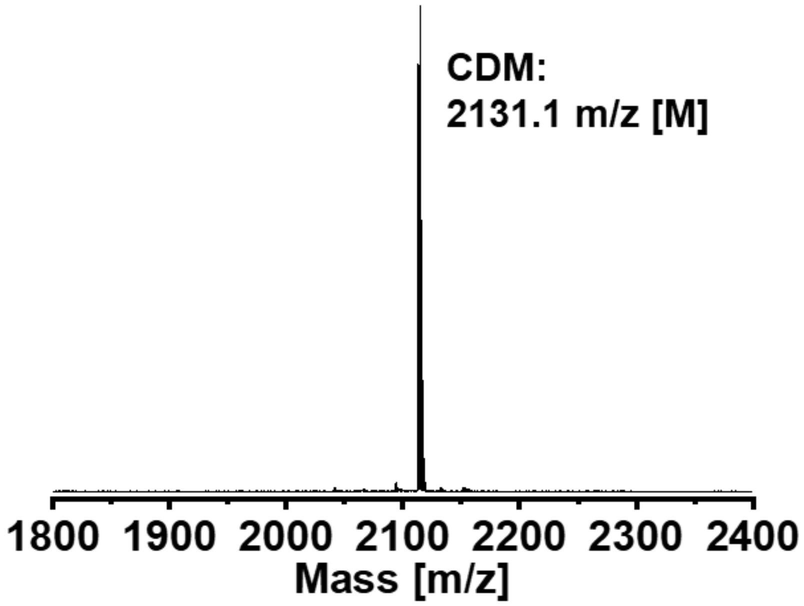

Figure S3. The exact molecular weight of CDM was calculated to be 2131.1 Da and measured to be 2131.1 m/z [M], which was confirmed via the MALDI-TOF mass spectrometer.

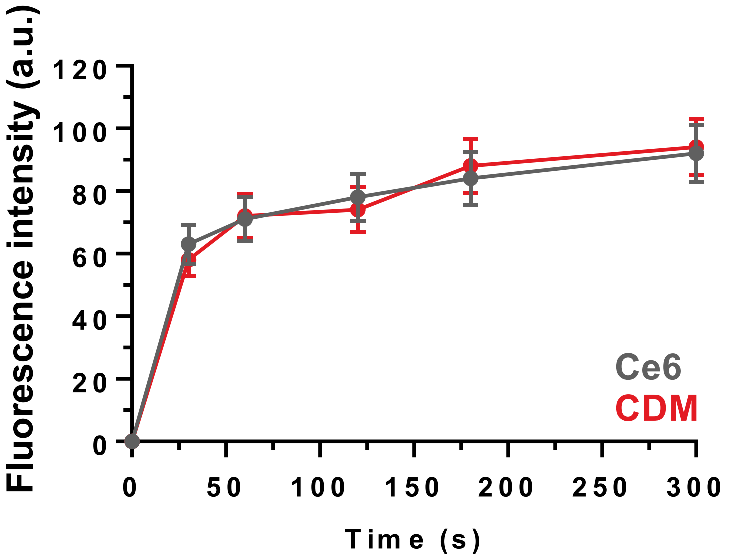

Figure S4. Singlet oxygen generation from Ce6 and CDM under visible light irradiation, as confirmed via the Singlet Oxygen Sensor Green (SOSG) assays.

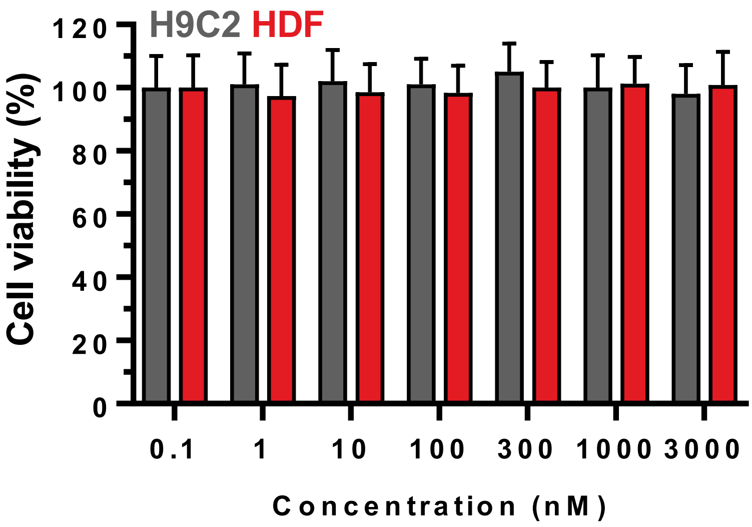

Figure S5. The viability of H9C2 and HDF cells after treatment of CDM in absence of visible light.

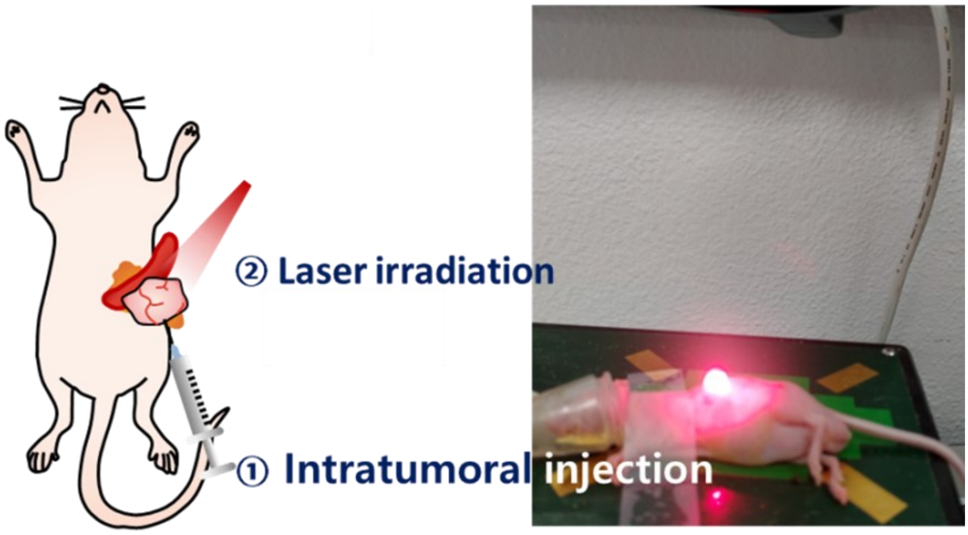

Figure S6. The photos to show the procedure of visible light irradiation for *in vivo* experiments.
